# A Uzawa-Type Iterative Algorithm for the Stationary Natural Convection Model

**DOI:** 10.3390/e24040543

**Published:** 2022-04-13

**Authors:** Aytura Keram, Pengzhan Huang

**Affiliations:** College of Mathematics and System Sciences, Xinjiang University, Urumqi 830017, China; 13899842064@stu.xju.edu.cn

**Keywords:** Uzawa algorithm, natural convection model, weakly divergence-free approximation, convergence

## Abstract

In this study, a Uzawa-type iterative algorithm is introduced and analyzed for solving the stationary natural convection model, where physical variables are discretized by utilizing a mixed finite element method. Compared with the common Uzawa iterative algorithm, the main finding is that the proposed algorithm produces weakly divergence-free velocity approximation. In addition, the convergence results of the proposed algorithm are provided, and numerical tests supporting the theory are presented.

## 1. Introduction

Arising both in nature and in engineering applications, the natural convection model is a coupled system of fluid flow governed by the incompressible Navier-Stokes equations and heat transfer governed by the energy equation. The natural convection problem has been a hot topic in heat transmission science for a long time, because it has been widely used in many fields of production and life, such as room ventilation, general heating, nuclear reaction systems, fire control, katabatic winds, atmospheric fronts, cooling of electronic equipment, natural ventilation, solar collectors, and so on [1,2,3]. In particular with nanofluids, the literature survey in [4] evidences the parameters governing the flow and heat behavior of fluids under natural convection and reveals that there are very few generalized correlations between heat transfer and wall heating conditions in enclosures.

Due to its practical significance, a considerable amount of researchers have put forward many efficient numerical methods to obtain the solution to this problem in different geometries [5,6,7,8,9,10]. For example, Boland and Layton [6,7] have proposed a Galerkin finite element method for the natural convection problem. Several iterative schemes based on the finite element method for the natural convection equations with different Rayleigh numbers have been studied in [9]. The coupled Navier-Stokes/temperature (or Boussinesq) equations [5] were solved by applying a divergence-free low order stabilized finite element method. A unified analysis approach of a local projection stabilization finite element method for solving natural convection problems was given by [8]. However, there still remain some important but challenging problems, especially solving the model effectively with the strong coupling between the velocity, pressure, and temperature fields and the saddle-point problem arising from finite element discretization.

As is known, the Uzawa method [11] is an efficient iterative algorithm for the saddle-point system. Since it is simple, efficient, and has minimal computer memory requirements, it has been widely used in computational science and engineering [12,13,14,15,16]. In particular, some Uzawa iterative methods were designed for the steady incompressible Navier-Stokes equations [17]. Further, the steady magnetohydrodynamic equations [18] and the steady natural convection equations [19] were solved by applying some Uzawa iterative algorithms. However, in these works, the weakly divergence-free constraint on the velocity was not enforced.

Recently, a Uzawa-type iterative algorithm [20] was designed for the coupled Stokes equations, where no saddle point system was required to be solved at each iteration step, and the weakly divergence-free velocity approximation was shown. Inspired by [20], in this article we propose and analyze a Uzawa-type iterative algorithm for the natural convection problem and obtain a numerical velocity, which satisfies the weakly divergence-free condition.

## 2. Preliminaries

Let $\Omega \subset R^{2}$ be a bounded domain, which has a Lipschitz continuous boundary ∂Ω with a regular open subset Γ. Consider the following stationary natural convection problem. Seek the velocity $u={\left(u_{1}\left(x\right),u_{2}\left(x\right)\right)}^{⊤}$, the pressure p=p(x), and the temperature T(x), such that

(1) $$\begin{array}{cccc} \nabla p+(u\cdot \nabla )u-Pr\Delta u & =PrRajT,\;\;\nabla \cdot u=0\; & \mathrm{in} & \;\Omega ,\; \end{array}$$

(2) $$\begin{array}{cccc} u & =0,\; & \mathrm{on} & \;\partial \Omega ,\; \end{array}$$

(3) $$\begin{array}{cccc} -\kappa \Delta T & =\gamma -u\cdot \nabla T,\; & \mathrm{in} & \;\Omega , \end{array}$$

(4) $$\begin{array}{cccc} T & =0,\;\mathrm{on}\;\partial \Gamma ,\;\frac{\partial T}{\partial n}=0,\; & \mathrm{on} & \;\partial \Omega \backslash \Gamma , \end{array}$$

where γ is the forcing function, n is the outward unit vector, and $j={\left(0,1\right)}^{⊤}$. In addition, the positive parameter κ presents the thermal conductivity, Pr is the Prandtl number, and Ra is the Rayleigh number.

Next, in order to write the variational form of (1)–(4), we introduce the following necessary function spaces:

$$\begin{array}{c} \begin{array}{cc} M & =H_{0}^{1}{\left(\Omega \right)}^{2}=\left\{v\in H^{1}{\left(\Omega \right)}^{2}:\;v=0\;\mathrm{on}\;\partial \Omega \right\},\\ W & =L_{0}^{2}\left(\Omega \right)=\left\{q\in L^{2}\left(\Omega \right):\;\left(q,1\right)=0\right\},\;Z=\left\{s\in H^{1}\left(\Omega \right):\;s=0\;\mathrm{on}\;\Gamma \right\}. \end{array} \end{array}$$

Here, the space $L^{2}\left(\Omega \right)$ is endowed with $L^{2}$-scalar product (·,·) and $L^{2}$-norm ∥·∥. In addition, the space $H^{1}\left(\Omega \right)$ is used to represent the standard definitions for Sobolev spaces $W^{m,p}\left(\Omega \right)$, m,p>0.

Moreover, we recall the Poincaré inequality [21] as follows:

(5) $$\begin{array}{c} \begin{array}{c} ∥v∥\leq C_{p}∥\nabla v∥,\;\;\forall v\in M, \end{array} \end{array}$$

where $C_{p}$ is the Poincaré constant. Next, we denote two trilinear forms by

$$\begin{array}{c} \begin{array}{c} b_{1}\left(u;v,w\right)=\left(\left(u\cdot \nabla \right)v,w\right)+\frac{1}{2}\left(\left(\nabla \cdot u\right)v,w\right),\;b_{2}\left(u;T,s\right)=\left(u\cdot \nabla T,s\right)+\frac{1}{2}\left(\left(\nabla \cdot u\right)T,s\right), \end{array} \end{array}$$

which satisfy the following properties [7,22,23]

(6) $$\begin{array}{c} \begin{array}{c} |b_{1}\left(u;v,w\right)|\leq N∥\nabla u∥∥\nabla v∥∥\nabla w∥,\;|b_{2}\left(u;T,s\right)|\leq \bar{N}∥\nabla u∥∥\nabla T∥∥\nabla s∥, \end{array} \end{array}$$

for all u,v,w,∈M and T,s∈Z. Here, *N* and $\bar{N}$ are two fixed positive constants.

With the above notations, the weak form of (1)–(4) reads as: find (u,p,T)∈M×W×Z such that

(7) $$\begin{array}{cc} \; & Pr\left(\nabla u,\nabla v\right)+b_{1}\left(u;u,v\right)-\left(p,\nabla \cdot v\right)=PrRa\left(jT,v\right),\;\forall v\in M, \end{array}$$

(8) $$\begin{array}{cc} \; & (\nabla \cdot u,q)=0,\;\forall q\in W, \end{array}$$

(9) $$\begin{array}{cc} \; & \kappa \left(\nabla T,\nabla s\right)+b_{2}\left(u;T,s\right)=\left(\gamma ,s\right),\;\forall s\in Z. \end{array}$$

The following existence and uniqueness of the solution to (6) are classical results.

**Theorem** **1** ([7,19])**.** *There exists at least a solution (u,p,T)∈M×W×Z, which satisfies *(7)–(9)* and*

$$\begin{array}{cc} ∥\nabla T∥ & \leq \kappa^{-1}{∥\gamma ∥}_{-1},\;∥\nabla u∥\leq C_{p}^{2}Ra\kappa^{-1}{∥\gamma ∥}_{-1}, \end{array}$$

*where ${∥\gamma ∥}_{-1}=\underset{s\in Z}{\sup}\frac{\left|(\gamma ,s)\right|}{∥\nabla s∥}.$ Further, if Pr, Ra, κ, and γ satisfy the uniqueness condition*

$$0<Pr^{-1}\Lambda +\bar{\Lambda }<1,$$

*where $\Lambda =C_{p}^{2}RaN\kappa^{-1}{∥\gamma ∥}_{-1}$ and $\bar{\Lambda }=C_{p}^{2}Ra\bar{N}\kappa^{-2}{∥\gamma ∥}_{-1}$, then the solution (u,p,T) of *(7)–(9)* is unique.*

Next, we consider a family of quasi-uniform and regular triangulations $K_{h}=\left\{K:\cup_{K\subset \Omega }\bar{K}=\bar{\Omega }\right\}$ with mesh size *h*, which is a partition of the domain Ω. Then, we assume that the finite element subspace $M_{h}\times W_{h}\times Z_{h}\subset M\times W\times Z$

$$M_{h}=\left\{v\in M\cap C^{0}{\left(\bar{\Omega }\right)}^{2}:v{|}_{K}\in P_{2}{\left(K\right)}^{2},\forall K\in K_{h}\right\},$$

$$W_{h}=\left\{q\in W\cap C^{0}\left(\bar{\Omega }\right):q{|}_{K}\in P_{1}\left(K\right),\forall K\in K_{h}\right\},$$

$$Z_{h}=\left\{s\in Z\cap C^{0}\left(\bar{\Omega }\right):s{|}_{K}\in P_{2}\left(K\right),\forall K\in K_{h}\right\},$$

where $P_{i}\left(K\right)$, i=1,2 is the set of all polynomials on *K* of a degree no more than *i*. As is known, the finite element subspaces $M_{h}\times W_{h}$ satisfy the following discrete inf-sup condition [21]; for each $q\in W_{h}$, there exists $v\in M_{h},v\neq 0$ such that $\inf_{q\in W_{h}}\sup_{v\in M_{h}}\frac{\left|(\nabla \cdot v,q)\right|}{∥\nabla v∥∥q∥}\geq \beta$, where the constant β∈(0,1] is proven in [24].

Moreover, according to the above definition of the finite element subspaces, the finite element approximation for (7)–(9) is to seek $\left(u_{h},p_{h},T_{h}\right)\in M_{h}\times W_{h}\times Z_{h}$ such that

(10) $$\begin{array}{cc} \; & Pr\left(\nabla u_{h},\nabla v\right)+b_{1}\left(u_{h};u_{h},v\right)-\left(p_{h},\nabla \cdot v\right)=PrRa\left(jT_{h},v\right),\;\forall v\in M_{h}, \end{array}$$

(11) $$\begin{array}{cc} \; & \left(\nabla \cdot u_{h},q\right)=0,\;\forall q\in W_{h}, \end{array}$$

(12) $$\begin{array}{cc} \; & \kappa \left(\nabla T_{h},\nabla s\right)+b_{2}\left(u_{h};T_{h},s\right)=\left(\gamma ,s\right),\;\forall s\in Z_{h}. \end{array}$$

The following theorem is established for the stability of the finite element discretization.

**Theorem** **2** ([6,9,25])**.** *Under the assumptions of Theorem 1, the finite element discretization *(10)–(12)* has at least a solution $\left(u_{h},p_{h},T_{h}\right)\in M_{h}\times W_{h}\times Z_{h}$, such that*

$$\begin{array}{c} ∥\nabla u_{h}∥\leq C_{p}^{2}Ra\kappa^{-1}{∥\gamma ∥}_{-1},\;∥\nabla T_{h}∥\leq \kappa^{-1}{∥\gamma ∥}_{-1}. \end{array}$$

## 3. A Uzawa-Type Iterative Algorithm

In this section, we present a Uzawa-type iterative algorithm for solving the considered problem. Before showing the algorithm, we recall the common Uzawa iterative algorithm based on the mixed finite element method as follows Algorithm 1.

According to the above algorithm, we find that $(\nabla \cdot u_{h}^{n+1},q)\neq 0$, which means that the divergence-free constraint on the velocity is not weakly enforced. In fact, from the finite element approximation (10)–(12), we have $(\nabla \cdot u_{h},q)=0$. Although it will result in a saddle problem, it produces weakly divergence-free velocity approximation. Hence, it is interesting to design a Uzawa-type iterative algorithm, which does not only retain the benefits of the common Uzawa iterative algorithm but also retains the velocity in a weakly divergence-free condition.
**Algorithm 1:** Uzawa iterative algorithm [19].**Step 1**. Find initial guess $\left(u_{h}^{0},p_{h}^{0},T_{h}^{0}\right)\in M_{h}\times W_{h}\times Z_{h}$ by
$$\begin{array}{c} \left\{\begin{array}{cc} & Pr\left(\nabla u_{h}^{0},\nabla v\right)-\left(p_{h}^{0},\nabla \cdot v\right)=PrRa\left(jT_{h}^{0},v\right),\;\;\forall v\in M_{h},\\ & \left(\nabla \cdot u_{h}^{0},q\right)=0,\;\forall q\in W_{h},\\ & \kappa \left(\nabla T_{h}^{0},\nabla s\right)=\left(\gamma ,s\right),\;\forall s\in Z_{h}. \end{array}\right. \end{array}$$**Step 2**. Given a relaxation parameter ρ>0, find $\left(u_{h}^{n+1},p_{h}^{n+1},T_{h}^{n+1}\right)\in M_{h}\times W_{h}\times Z_{h}$ as solution of
$$\begin{array}{c} \left\{\begin{array}{cc} & Pr\left(\nabla u_{h}^{n+1},\nabla v\right)+b_{1}\left(u_{h}^{n};u_{h}^{n+1},v\right)-\left(p_{h}^{n},\nabla \cdot v\right)=PrRa\left(jT_{h}^{n+1},v\right),\;\;\forall v\in M_{h},\\ & \left(p_{h}^{n+1},q\right)=\left(p_{h}^{n},q\right)-\rho \left(\nabla \cdot u_{h}^{n+1},q\right),\;\forall q\in W_{h},\\ & \kappa \left(\nabla T_{h}^{n+1},\nabla s\right)+b_{2}\left(u_{h}^{n};T_{h}^{n+1},s\right)=\left(\gamma ,s\right),\;\forall s\in Z_{h}. \end{array}\right. \end{array}$$

In order to make the velocity of Uzawa algorithm have a weakly divergence-free property, let *g* be a gauge variable [26] and d be a variable, such that u=d+∇g. If *g* and *p* satisfy an elliptic equation PrΔg=p, then (1)–(4) can be rewritten as

$$\begin{array}{c} \begin{array}{cc} -Pr\Delta d+((d+\nabla g)\cdot \nabla )(d+\nabla g) & =PrRajT,\\ \nabla \cdot d & =-\Delta g,\\ -\kappa \Delta T+(d+\nabla g)\cdot \nabla T & =\gamma . \end{array} \end{array}$$

Furthermore, begin with $g^{0}=g^{-1}=0$ and $d^{0}=u_{h}^{0}$. Repeat

(13) $$\begin{array}{cc} -Pr\Delta d^{n+1}+\left(\left(d^{n}+\nabla g^{n-1}\right)\cdot \nabla \right)\left(d^{n+1}+\nabla g^{n}\right) & =PrRajT^{n+1}, \end{array}$$

(14) $$\begin{array}{cc} \nabla \cdot d^{n+1} & =-\Delta g^{n+1},\; \end{array}$$

(15) $$\begin{array}{cc} -\kappa \Delta T^{n+1}+\left(d^{n}+\nabla g^{n-1}\right)\cdot \nabla T^{n+1} & =\gamma , \end{array}$$

for n=0,1,…

Moreover, setting ${\hat{u}}^{n+1}=d^{n+1}+\nabla g^{n}$ in (13)–(15), we have

(16) $$\begin{array}{cc} -Pr\Delta {\hat{u}}^{n+1}+\left({\hat{u}}^{n}\cdot \nabla \right){\hat{u}}^{n+1}+\nabla p^{n} & =PrRajT^{n+1}, \end{array}$$

(17) $$\begin{array}{cc} \nabla \cdot {\hat{u}}^{n+1} & =-\Delta \hbar^{n+1},\; \end{array}$$

(18) $$\begin{array}{cc} -\nabla \cdot \left(\kappa \nabla T^{n+1}\right)+\left({\hat{u}}^{n}\cdot \nabla \right)T^{n+1} & =\gamma , \end{array}$$

where $\hbar^{n+1}:=g^{n+1}-g^{n}$. So one obtains

$$p^{n+1}=P_{r}\Delta g^{n+1}=P_{r}\Delta \hbar^{n+1}+Pr\Delta g^{n}=P_{r}\Delta \hbar^{n+1}+p^{n},$$

and

$$u^{n+1}=d^{n+1}+\nabla g^{n+1}={\hat{u}}^{n+1}-\nabla g^{n}+\nabla g^{n+1}={\hat{u}}^{n+1}+\nabla \hbar^{n+1}.$$

Now, we are ready to write the Uzawa-type finite element iterative algorithm as follows Algorithm 2.
**Algorithm 2:** Uzawa-type iterative algorithm.**Step 1**. Obtain the initial guess $\left(u_{h}^{0},p_{h}^{0},T_{h}^{0}\right)\in M_{h}\times W_{h}\times Z_{h}$ from step 1 of Algorithm 1.**Step 2**. Find $\left({\hat{u}}_{h}^{n+1},T_{h}^{n+1}\right)\in M_{h}\times Z_{h}$ as the solution of
(19)$$\begin{array}{cc} \; & \kappa \left(\nabla T_{h}^{n+1},\nabla s\right)+b_{2}\left({\hat{u}}_{h}^{n};T_{h}^{n+1},s\right)=\left(\gamma ,s\right),\;\forall s\in Z_{h}, \end{array}$$
(20)$$\begin{array}{cc} \; & Pr\left(\nabla {\hat{u}}_{h}^{n+1},\nabla v\right)+b_{1}\left({\hat{u}}_{h}^{n};{\hat{u}}_{h}^{n+1},v\right)-\left(p_{h}^{n},\nabla \cdot v\right)=PrRa\left(jT_{h}^{n+1},v\right),\;\;\forall v\in M_{h}. \end{array}$$**Step 3**. Find $\hbar_{h}^{n+1}\in W_{h}$ as the solution of
(21)$$\begin{array}{c} \begin{array}{c} \left(\nabla \hbar_{h}^{n+1},\nabla q\right)=\left(\nabla \cdot {\hat{u}}_{h}^{n+1},q\right),\;q\in W_{h}. \end{array} \end{array}$$**Step 4**. Compute $u_{h}^{n+1}$ with $u_{h}^{n+1}={\hat{u}}_{h}^{n+1}+\nabla \hbar_{h}^{n+1}$.**Step 5**. Given a relaxation parameter ρ>0, find $p_{h}^{n+1}\in W_{h}$ from the Richardson update
(22)$$\begin{array}{c} \begin{array}{c} \left(p_{h}^{n+1},q\right)=\left(p_{h}^{n},q\right)-Pr\rho \left(\nabla \hbar_{h}^{n+1},\nabla q\right),\;\forall q\in W_{h}. \end{array} \end{array}$$From (21) and Step 4 of Algorithm 2, we obtain $\left(\nabla \cdot u_{h}^{n+1},q\right)=\left(\nabla \cdot {\hat{u}}_{h}^{n+1},q\right)-\left(\nabla \hbar_{h}^{n+1},\nabla q\right)=0$. So the velocity obtained by Algorithm 2 satisfies the weakly divergence-free condition. Moreover, we expect to show the iterative errors between the finite element solutions to (10)–(12) and the Uzawa-type iterative solutions to Algorithm 2. For convenience, assume that $E_{h}^{n}=u_{h}-u_{h}^{n}$, ${\hat{E}}_{h}^{n}=u_{h}-{\hat{u}}_{h}^{n}$, $\eta_{h}^{n}=p_{h}-p_{h}^{n}$ and $\theta_{h}^{n}=T_{h}-T_{h}^{n}$. Then, we have ${\hat{E}}_{h}^{n}=E_{h}^{n}+\nabla \hbar_{h}^{n}$.

Firstly, we recall the convergence results of the initial guess. Note that ${\hat{u}}_{h}^{0}=d^{0}+\nabla g^{-1}=u_{h}^{0}$, which implies $E_{h}^{0}={\hat{E}}_{h}^{0}$.

**Lemma** **1** ([19])**.** *Let $\left(u_{h}^{0},p_{h}^{0},T_{h}^{0}\right)\in M^{h}\times W^{h}\times Z^{h}$ be the solution of Step 1 of Algorithm 1. Then, under the assumptions of Theorem 2, we have the following results*

$$\begin{array}{cc} ∥\nabla \theta_{h}^{0}∥ & \leq k^{-1}\bar{\Lambda }{∥\gamma ∥}_{-1},\;∥\eta_{h}^{0}∥\leq 2\beta^{-1}Pr\Lambda N^{-1}\left(Pr^{-1}\Lambda +\bar{\Lambda }\right),\;∥\nabla E_{h}^{0}∥\leq \Lambda N^{-1}\left(Pr^{-1}\Lambda +\bar{\Lambda }\right). \end{array}$$

Secondly, we show that the solution sequence generated by Algorithm 2 is bounded.

**Theorem** **3.** *Let $\left\{u_{h}^{n},p_{h}^{n},T_{h}^{n}\right\}$ be the solution sequence of Algorithm 2. Then, under the assumptions of Theorem 2, if the relaxation parameter satisfies $\rho \in (0,2\left(1-\bar{\Lambda }-Pr^{-1}\Lambda \right))$, the sequences $\left\{∥\nabla u_{h}^{n}∥\right\}$, $\left\{∥\nabla {\hat{u}}_{h}^{n}∥\right\}$, $\left\{∥p_{h}^{n}∥\right\}$ and $\left\{∥\nabla T_{h}^{n}∥\right\}$ are uniformly bounded with respect to h.*

**Proof.**  Subtracting (19) from (12), we have

$$\begin{array}{c} \begin{array}{c} b_{2}\left({\hat{E}}_{h}^{n};T_{h},s\right)-b_{2}\left({\hat{u}}_{h}^{n};\theta_{h}^{n+1},s\right)+\kappa \left(\nabla \theta_{h}^{n+1},\nabla s\right)=0. \end{array} \end{array}$$

Setting $s=\theta_{h}^{n+1}$ obtains

$$\kappa ∥\nabla \theta_{h}^{n+1}{∥}^{2}=-b_{2}\left({\hat{E}}_{h}^{n};T_{h},\theta_{h}^{n+1}\right).$$

According to (6) and Theorem 2, we arrive at

(23) $$\begin{array}{c} \begin{array}{c} ∥\nabla \theta_{h}^{n+1}∥\leq \bar{N}\kappa^{-2}{∥\gamma ∥}_{-1}∥\nabla {\hat{E}}_{h}^{n}∥. \end{array} \end{array}$$

Then, subtracting (20) from (10), we have

(24) $$\begin{array}{c} \begin{array}{c} Pr\left(\nabla {\hat{E}}_{h}^{n+1},\nabla v\right)-\left(\eta_{h}^{n},\nabla \cdot v\right)=-b_{1}\left({\hat{E}}_{h}^{n};u_{h},v\right)-b_{1}\left({\hat{u}}_{h}^{n};{\hat{E}}_{h}^{n+1},v\right)+PrRa\left(j\theta_{h}^{n+1},v\right). \end{array} \end{array}$$

Choosing $v={\hat{E}}_{h}^{n+1}$ in (24) and combining the ensuing equation with (21) lead to

$$\begin{array}{c} Pr∥\nabla {\hat{E}}_{h}^{n+1}{∥}^{2}=-\left(\nabla \eta_{h}^{n},\nabla \hbar_{h}^{n+1}\right)-b_{1}\left({\hat{E}}_{h}^{n};u_{h},{\hat{E}}_{h}^{n+1}\right)+PrRa\left(j\theta_{h}^{n+1},{\hat{E}}_{h}^{n+1}\right). \end{array}$$

Next, according to (22), we have

$$\begin{array}{c} \begin{array}{c} Pr∥\nabla {\hat{E}}_{h}^{n+1}{∥}^{2}={\left(Pr\rho \right)}^{-1}\left(p_{h}^{n+1}-p_{h}^{n},\eta_{h}^{n}\right)-b_{1}\left({\hat{E}}_{h}^{n};u_{h},{\hat{E}}_{h}^{n+1}\right)+PrRa\left(j\theta_{h}^{n+1},{\hat{E}}_{h}^{n+1}\right), \end{array} \end{array}$$

which, by using (5), (6), (23), Theorem 2, and the Proposition identity $\left(u,v\right)=\frac{1}{2}{(∥u+v∥}^{2}-{∥u∥}^{2}-{∥v∥}^{2})$, we have

(25) $$\begin{array}{c} \begin{array}{cc} 2Pr^{2}\rho ∥\nabla {\hat{E}}_{h}^{n+1}{∥}^{2}+{∥\eta_{h}^{n+1}∥}^{2}\leq & ∥\eta_{h}^{n}{∥}^{2}+{∥\eta_{h}^{n+1}-\eta_{h}^{n}∥}^{2}\\ & +2Pr\rho \left(\Lambda +Pr\bar{\Lambda }\right)∥\nabla {\hat{E}}_{h}^{n}∥∥\nabla {\hat{E}}_{h}^{n+1}∥. \end{array} \end{array}$$

Then, using (21) and (22), we obtain

$$\begin{array}{c} \begin{array}{cc} ∥\eta_{h}^{n+1}-\eta_{h}^{n}{∥}^{2} & =\left(p_{h}^{n+1}-p_{h}^{n},p_{h}^{n+1}-p_{h}^{n}\right)=-Pr\rho \left(\nabla \hbar_{h}^{n+1},\nabla \left(\eta_{h}^{n+1}-\eta_{h}^{n}\right)\right)\\ & =Pr\rho (\nabla \cdot {\hat{E}}_{h}^{n+1},\eta_{h}^{n+1}-\eta_{h}^{n}), \end{array} \end{array}$$

which leads to

(26) $$\begin{array}{c} \begin{array}{c} ∥\eta_{h}^{n+1}-\eta_{h}^{n}{∥}^{2}\leq {\left(Pr\rho \right)}^{2}∥\nabla \cdot {\hat{E}}_{h}^{n+1}{∥}^{2}\leq {\left(Pr\rho \right)}^{2}{∥\nabla {\hat{E}}_{h}^{n+1}∥}^{2}, \end{array} \end{array}$$

where we have applied the fact that ∥∇·v∥≤∥∇v∥ in [24].

Moreover, substituting (26) into (25) and using the Young inequality, we obtain

(27) $$\begin{array}{c} \begin{array}{cc} & \;∥\nabla {\hat{E}}_{h}^{n+1}{∥}^{2}\left(2Pr^{2}\rho -Pr^{2}\rho^{2}-\varsigma \left(Pr\rho \Lambda +Pr^{2}\rho \bar{\Lambda }\right)\right)+{∥\eta_{h}^{n+1}∥}^{2}\\ & \leq ∥\eta_{h}^{n}{∥}^{2}+\varsigma^{-1}\left(Pr\rho \Lambda +Pr^{2}\rho \bar{\Lambda }\right){∥\nabla {\hat{E}}_{h}^{n}∥}^{2}, \end{array} \end{array}$$

where ς>0 is a parameter to be determined later on.

Furthermore, we solve a quadratic algebraic equation

$$\varsigma^{2}\left(\Lambda +Pr\bar{\Lambda }\right)-\varsigma \left(2Pr-Pr\rho \right)+\left(\Lambda +Pr\bar{\Lambda }\right)=0,$$

to obtain a positive root $\varsigma =\varsigma^{*}$, which makes $\left(2Pr-Pr\rho -\varsigma \left(\Lambda +Pr\bar{\Lambda }\right)\right)=\varsigma^{-1}\left(\Lambda +Pr\bar{\Lambda }\right)$ hold. In fact, we have

$$\varsigma =\varsigma^{*}=\frac{\left(2Pr-Pr\rho \right)-\sqrt{\Delta }}{2(\Lambda +Pr\bar{\Lambda })},$$

where $\Delta :=\left(2Pr-Pr\rho +2\left(\Lambda +Pr\bar{\Lambda }\right)\right)\left(2Pr-Pr\rho -2\left(\Lambda +Pr\bar{\Lambda }\right)\right)$.

Next, we set

$$\begin{array}{c} D_{1}=Pr\rho \left(2Pr-Pr\rho -\varsigma^{*}\left(\Lambda +Pr\bar{\Lambda }\right)\right)=Pr\rho \left(\Lambda +Pr\bar{\Lambda }\right)/\varsigma^{*}=\frac{Pr^{2}\rho \left(2-\rho \right)+\sqrt{\Delta }}{2}. \end{array}$$

Thus, the inequality (27) is rewritten as

$$D_{1}∥\nabla {\hat{E}}_{h}^{n+1}{∥}^{2}+∥\eta_{h}^{n+1}{∥}^{2}\leq ∥\eta_{h}^{n}{∥}^{2}+D_{1}{∥\nabla {\hat{E}}_{h}^{n}∥}^{2},$$

which, along with (23), implies that

(28) $$\begin{array}{c} \begin{array}{cc} D_{1}∥\nabla {\hat{E}}_{h}^{n+1}{∥}^{2}+{∥\eta_{h}^{n+1}∥}^{2} & \leq ∥\eta_{h}^{0}{∥}^{2}+D_{1}{∥\nabla {\hat{E}}_{h}^{0}∥}^{2},\\ ∥\nabla \theta_{h}^{n+1}∥ & \leq {\bar{N}}^{2}\kappa^{-4}{∥\gamma ∥}_{-1}^{2}(∥\eta_{h}^{0}{∥}^{2}+D_{1}∥\nabla {\hat{E}}_{h}^{0}{∥}^{2}). \end{array} \end{array}$$

Finally, applying (26) into (22), we obtain

$$\begin{array}{c} \begin{array}{c} ∥\nabla \hbar_{h}^{n+1}∥\leq C_{p}^{2}{\left(Pr\rho \right)}^{-1}∥p_{h}^{n}-p_{h}^{n+1}∥\leq C_{p}^{2}{\left(Pr\rho \right)}^{-1}∥\eta_{h}^{n+1}-\eta_{h}^{n}∥\leq C_{p}^{2}∥\nabla {\hat{E}}_{h}^{n+1}∥, \end{array} \end{array}$$

which combines with ${\hat{E}}_{h}^{n+1}=E_{h}^{n+1}+\nabla \hbar_{h}^{n+1}$; then, we have

(29) $$\begin{array}{c} \begin{array}{c} ∥E_{h}^{n+1}{∥}^{2}\leq 2(∥{\hat{E}}_{h}^{n+1}{∥}^{2}+∥\nabla \hbar_{h}^{n+1}{∥}^{2})\leq 4C_{p}^{4}{∥\nabla {\hat{E}}_{h}^{n+1}∥}^{2}, \end{array} \end{array}$$

Finally, combining (29) with (28), we obtain

(30) $$\begin{array}{c} \begin{array}{c} D_{1}∥E_{h}^{n+1}{∥}^{2}\leq 4C_{p}^{4}(∥\eta_{h}^{0}{∥}^{2}+D_{1}∥\nabla {\hat{E}}_{h}^{0}{∥}^{2}). \end{array} \end{array}$$

Hence, using (28), (30), and Lemma 1, we finish the proof of the theorem. □

Thirdly, we are going to develop the convergence analysis for Algorithm 2.

**Theorem** **4.** *Under the assumptions of Theorem 3, the following estimates hold*

$$\begin{array}{cc} Pr^{2}D{∥E_{h}^{n+1}∥}^{2} & \leq 4C_{p}^{4}H^{n+1}(Pr^{2}D∥\nabla {\hat{E}}_{h}^{0}{∥}^{2}+∥\eta_{h}^{0}{∥}^{2}),\;∥\eta_{h}^{n+1}{∥}_{0}^{2}\leq H^{n+1}(Pr^{2}D∥\nabla {\hat{E}}_{h}^{0}{∥}^{2}+∥\eta_{h}^{0}{∥}^{2}),\\ Pr^{2}D{∥\nabla \theta_{h}^{n+1}∥}^{2} & \leq {\bar{N}}^{2}\kappa^{-4}{∥\gamma ∥}_{-1}^{2}H^{n}(Pr^{2}D∥\nabla {\hat{E}}_{h}^{0}{∥}^{2}+∥\eta_{h}^{0}{∥}^{2}), \end{array}$$

*where $D\in (0,\frac{1}{2})$ and $H\in (\frac{3}{4},1)$ are two constants independent of n and h.*

**Proof.**  By Theorem 3, there exists a positive constant $D_{2}$, independent of *n* and *h*, such that

(31) $$\begin{array}{c} ∥\nabla {\hat{u}}_{h}^{n}∥\leq D_{2}. \end{array}$$

Then, rewrite (24) to obtain

$$\begin{array}{c} \left(\eta_{h}^{n},\nabla \cdot v\right)=Pr\left(\nabla {\hat{E}}_{h}^{n+1},\nabla v\right)+b_{1}\left({\hat{E}}_{h}^{n};u_{h},v\right)+b_{1}\left({\hat{u}}_{h}^{n};{\hat{E}}_{h}^{n+1},v\right)-PrRa\left(j\theta_{h}^{n+1},v\right). \end{array}$$

Applying the inf-sup condition, (5), (6), (23), and Theorem 2 to the above equation, we obtain

$$\begin{array}{cc} \beta ∥\eta_{h}^{n}∥\leq & Pr∥\nabla {\hat{E}}_{h}^{n+1}∥+PrRaC_{p}^{2}\bar{N}\kappa^{-2}{∥\gamma ∥}_{-1}∥\nabla {\hat{E}}_{h}^{n}∥+PrRaC_{p}^{2}N\kappa^{-1}{∥\gamma ∥}_{-1}∥\nabla {\hat{E}}_{h}^{n}∥\\ \; & +N∥\nabla {\hat{u}}_{h}^{n}∥∥\nabla {\hat{E}}_{h}^{n+}∥, \end{array}$$

which combines with (31) to obtain

$$\begin{array}{c} \beta ∥\eta_{h}^{n}∥\leq \left(Pr+ND_{2}\right)∥\nabla {\hat{E}}_{h}^{n+1}∥+\left(\Lambda +Pr\bar{\Lambda }\right)∥\nabla {\hat{E}}_{h}^{n}∥. \end{array}$$

Next, using the inequality ${\left(a+b\right)}^{2}\leq 2a^{2}+2b^{2}$, we have

$$\begin{array}{c} \beta^{2}∥\eta_{h}^{n}{∥}^{2}\leq 2{\left(Pr+ND_{2}\right)}^{2}∥\nabla {\hat{E}}_{h}^{n+1}{∥}^{2}+2{\left(\Lambda +Pr\bar{\Lambda }\right)}^{2}{∥\nabla {\hat{E}}_{h}^{n}∥}^{2}. \end{array}$$

Hence, one obtains

(32) $$\begin{array}{c} \begin{array}{c} ∥\nabla {\hat{E}}_{h}^{n+1}{∥}^{2}\geq D_{3}∥\eta_{h}^{n}{∥}^{2}-D_{4}{∥\nabla {\hat{E}}_{h}^{n}∥}^{2}, \end{array} \end{array}$$

where $D_{3}:=\frac{\beta^{2}}{2{\left(Pr+ND_{2}\right)}^{2}}$ and $D_{4}:=\frac{{\left(\Lambda +Pr\bar{\Lambda }\right)}^{2}}{{\left(Pr+ND_{2}\right)}^{2}}$. Obviously, if we let $C_{\rho ,\varsigma }:=Pr\rho \left(2Pr-Pr\rho -\varsigma \left(\Lambda +Pr\bar{\Lambda }\right)\right)$, then (27) becomes

(33) $$\begin{array}{c} \begin{array}{c} \delta ∥\nabla {\hat{E}}_{h}^{n+1}{∥}^{2}+\left(C_{\rho ,\varsigma }-\delta \right)∥\nabla {\hat{E}}_{h}^{n+1}{∥}^{2}+∥\eta_{h}^{n+1}{∥}^{2}\leq ∥\eta_{h}^{n}{∥}^{2}+\varsigma^{-1}\left(Pr\rho \Lambda +Pr^{2}\rho \bar{\Lambda }\right){∥\nabla {\hat{E}}_{h}^{n}∥}^{2}. \end{array} \end{array}$$

where $\delta \in (0,C_{\rho ,\varsigma })$ is a parameter to be determined. From (32) and (33), we obtain

(34) $$\begin{array}{c} \begin{array}{cc} & \;\left(C_{\rho ,\varsigma }-\delta \right)∥\nabla {\hat{E}}_{h}^{n+1}{∥}^{2}+{∥\eta_{h}^{n+1}∥}^{2}\\ & \leq \left(1-D_{3}\delta \right)∥\eta_{h}^{n}{∥}^{2}+\left(\varsigma^{-1}\left(Pr\rho \Lambda +Pr^{2}\rho \bar{\Lambda }\right)+D_{4}\delta \right){∥\nabla {\hat{E}}_{h}^{n}∥}^{2}. \end{array} \end{array}$$

Then, we will choose parameters ς and δ such that

(35) $$\begin{array}{c} \begin{array}{c} \frac{C_{\rho ,\varsigma }-\delta }{1}=\frac{\varsigma^{-1}Pr\rho \left(\Lambda +Pr\bar{\Lambda }\right)+D_{4}\delta }{1-\delta D_{3}}, \end{array} \end{array}$$

and $1-\delta D_{3}>0$, which leads to

(36) $$\begin{array}{c} \begin{array}{c} D_{3}\delta^{2}-\left(1+C_{\rho ,\varsigma }D_{3}+D_{4}\right)\delta +C_{\rho ,\varsigma }-\varsigma^{-1}Pr\rho \left(\Lambda +Pr\bar{\Lambda }\right)=0. \end{array} \end{array}$$

In fact, one finds that

$$C_{\rho ,\varsigma }-\varsigma^{-1}Pr\rho \left(\Lambda +Pr\bar{\Lambda }\right)=\left(1+C_{\rho ,\varsigma }D_{3}+D_{4}\right)\delta -D_{3}\delta^{2}>C_{\rho ,\varsigma }D_{3}\delta -D_{3}\delta^{2}>0,$$

which, along with the definition of $C_{\rho ,\varsigma }$, yields

$$\left(\Lambda +Pr\bar{\Lambda }\right)\varsigma^{2}-\left(2Pr-Pr\rho \right)\varsigma +\left(\Lambda +Pr\bar{\Lambda }\right)<0,$$

and

$$\frac{\left(2Pr-Pr\rho \right)-\sqrt{\Delta }}{2(\Lambda +Pr\bar{\Lambda })}<\varsigma <\frac{\left(2Pr-Pr\rho \right)+\sqrt{\Delta }}{2(\Lambda +Pr\bar{\Lambda })},$$

where the notation Δ is defined in the proof of Theorem 3. Note that we have used condition $0<\rho <2(1-\bar{\Lambda }-Pr^{-1}\Lambda )$. Here, we select

$$\varsigma =\varsigma^{+}=\frac{2Pr-Pr\rho }{2(\Lambda +Pr\bar{\Lambda })}.$$

Substituting this parameter into (36), we arrive at $a\delta^{2}-b\delta +c=0$, where $a=D_{3}$, $b=1+D_{4}+s_{1}a$, $c=s_{1}-\frac{Pr^{2}\rho^{2}{\left(\Lambda +Pr\bar{\Lambda }\right)}^{2}}{s_{1}}$, and $s_{1}=Pr^{2}\rho \left(1-\frac{1}{2}\rho \right)$. Obviously, $b>1+s_{1}a$, $c<s_{1}$; so, we deduce that

$$b^{2}-4ac>{\left(1+s_{1}a\right)}^{2}-4as_{1}\geq 0.$$

Then, the Equation (36) has a real root $\delta^{*}=\frac{b-\sqrt{b^{2}-4ac}}{2a}$.

With the parameter ε and δ given by $\varepsilon^{+}$ and $\delta^{*}$, it follows from (34) that

(37) $$\begin{array}{c} \begin{array}{c} \bar{D}∥\nabla {\hat{E}}_{h}^{n+1}{∥}^{2}+∥\eta_{h}^{n+1}{∥}_{0}^{2}\leq H(\bar{D}∥\nabla {\hat{E}}_{h}^{n}{∥}^{2}+∥\eta_{h}^{n}{∥}^{2}), \end{array} \end{array}$$

where $\bar{D}=s_{1}-\delta^{*}$ and $H=1-\delta^{*}D_{3}$.

Note that $\bar{D}>0$ and H>0. Now, we will prove them. Consider the quadratic function $f\left(\delta \right)=a\delta^{2}-b\delta +c$. Because a>0, $s_{1}>0$, $b>1+s_{1}a$ and $c<s_{1}$, we obtain $\lim_{\delta \to -\infty }f\left(\delta \right)=\infty$ and

$$f\left(s\right)=as_{1}^{2}-bs_{1}+c<as_{1}^{2}-\left(1+as_{1}\right)s_{1}+s_{1}=0.$$

Thus, the smallest root $\delta^{*}$ of f(δ) must belong to $\left(-\infty ,s_{1}\right)$. So, the inequality $\bar{D}>0$ holds. Noticing that $C_{\rho ,\varsigma^{+}}-\delta^{*}=s_{1}-\delta^{*}>0$, it follows readily from (35) that H>0.

Finally, note that $0<\bar{D}<s_{1}=Pr^{2}\rho -\frac{1}{2}Pr^{2}\rho^{2}\leq \frac{Pr^{2}}{2}$. If, we choose the $\bar{D}=Pr^{2}D$ and $0<D<\frac{1}{2}$, the inequality (37) is rewritten as

(38) $$\begin{array}{c} Pr^{2}D∥\nabla {\hat{E}}_{h}^{n+1}{∥}^{2}+∥\eta_{h}^{n+1}{∥}_{0}^{2}\leq H_{1}(Pr^{2}D∥\nabla {\hat{E}}_{h}^{n}{∥}^{2}+∥\eta_{h}^{n}{∥}^{2}). \end{array}$$

According to the definition of $D_{3}$ and β≤1, we arrive at $D_{3}\leq \frac{1}{2Pr^{2}}$. Noticing that $\delta^{*}<s_{1}<\frac{Pr^{2}}{2}$, we easily find that $1>H=1-\delta^{*}D_{3}>\frac{3}{4}$.

Next, using (38) and (29), we obtain

$$\begin{array}{c} Pr^{2}D∥E_{h}^{n+1}{∥}^{2}\leq 4C_{p}^{4}H^{n+1}(Pr^{2}D∥\nabla {\hat{E}}_{h}^{0}{∥}^{2}+∥\eta_{h}^{0}{∥}^{2}),\;∥\eta_{h}^{n+1}{∥}_{0}^{2}\leq H^{n+1}(Pr^{2}D∥\nabla {\hat{E}}_{h}^{0}{∥}^{2}+∥\eta_{h}^{0}{∥}^{2}). \end{array}$$

Finally, using the above estimates with (23), we finish the proof. □

## 4. Numerical Study

We will represent some numerical tests to claim the accuracy and performance of the proposed algorithm for the steady natural convection problem in this section. We used the public finite element software FreeFem++ [27] and applied $P_{2}-P_{1}-P_{2}$ element to approximate the velocity, temperature, and pressure, respectively.

In the first numerical test, let the domain Ω=[0,1]×[0,1], and the right-hand side of (1)–(4) is selected such that the exact solutions are given by

$$\begin{array}{cc} p(x,y) & =\cos\left(\pi x\right)\cos\left(\pi y\right),\;T\left(x,y\right)=u_{1}\left(x,y\right)+u_{2}\left(x,y\right)\\ u_{1}\left(x,y\right) & =2\pi \sin^{2}\left(\pi x\right)\sin\left(\pi y\right)\cos\left(\pi y\right),\;u_{2}\left(x,y\right)=-2\pi \sin\left(\pi x\right)\sin^{2}\left(\pi y\right)\cos\left(\pi x\right). \end{array}$$

Here, we set the parameters Ra=Pr=κ=1 and use the stopping rule

$$\max\left\{\frac{∥u_{h}^{n+1}-u_{h}^{n}∥}{∥u_{h}^{n}∥},\frac{∥p_{h}^{n+1}-p_{h}^{n}∥}{∥p_{h}^{n}∥},\frac{∥T_{h}^{n+1}-T_{h}^{n}∥}{∥T_{h}^{n}∥}\right\}<1.0\times 10^{-6}.$$

**Figure 1:**
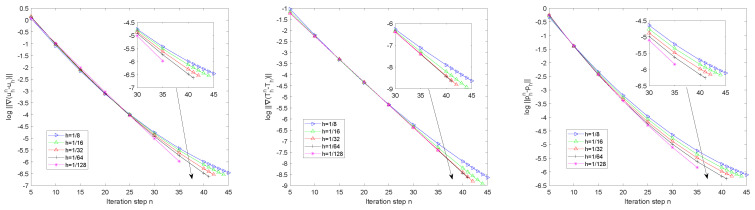
The log errors for different iterative steps *n* and different mesh sizes h.

Figure 1 displays the iteration errors of the velocity, temperature in $H^{1}$-seminorm, and the pressure in $L^{2}$-norm for different iterative steps *n* solved by Algorithm 2. Here, we set the relaxation parameter ρ=1.6 and choose five different mesh sizes *h*. From Figure 1, we observe that the proposed algorithm worked well and kept the convergence when iteration step *n* became large.

**Figure 2:**
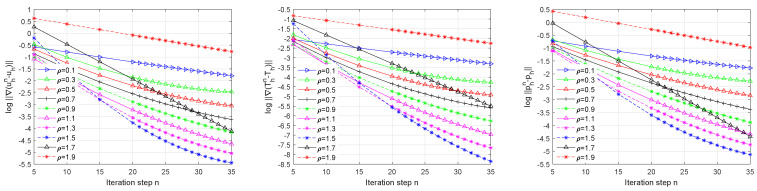
The log errors for different iterative steps *n* for different relaxation parameters ρ.

In the above test, we fixed the relaxation parameter and varied the mesh size. Now, we consider different relaxation parameters with the mesh size $h=\frac{1}{32}$. Figure 2 expresses different iterative steps of the log errors with different values ρ. From Figure 2, we observe that $u_{h}^{n}$, $p_{h}^{n}$, and $T_{h}^{n}$ converged faster when ρ was larger. However, we have an interesting observation that it became slow when ρ was too large (e.g., ρ=1.7 or 1.9). It is not surprising since from Theorem 3 and 4 the relaxation parameter ρ had a limited interval, and the value ρ=1.7 or 1.9 may have been out of its interval.

Hence, we should reveal the convergence on the relaxation parameter ρ by showing the values with respect to *n* and ρ under the mesh size $h=\frac{1}{32}$. From Table 1, we find that Algorithms 1 and 2 converged faster when we chose larger ρ. However, if the ρ chosen was very large, then these algorithms either need more iterative steps or diverge. In addition, Algorithms 1 and 2 achieved the tolerance error when ρ=1.6 with the least iterative steps n=44 and n=42, respectively.

Based on the previous section, Algorithm 2 produced the divergence-free velocity approximation. Hence, in Table 2 we list the value of $∥\nabla \cdot u_{h}^{n}∥$. From this table, Algorithms 1 and 2 obtain good numerical results when Ra=10. However, when the value of Ra increased, then Algorithm 1 could not achieve the tolerance error and converge. Meanwhile, Algorithm 2 still ran well.

**Figure 3:**
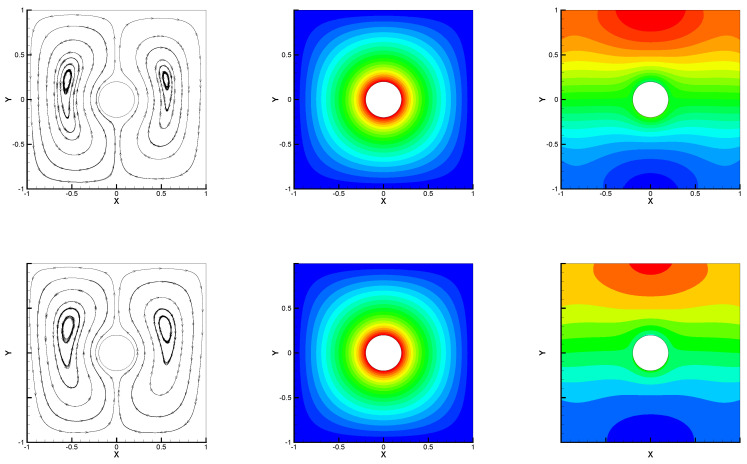
Numerical streamlines (the first column), isotherms (the second column), and isobars (the third column) for Ra=100 (the first line) and Ra=250 (the second line) with $r_{in}=0.2$.

**Figure 4:**
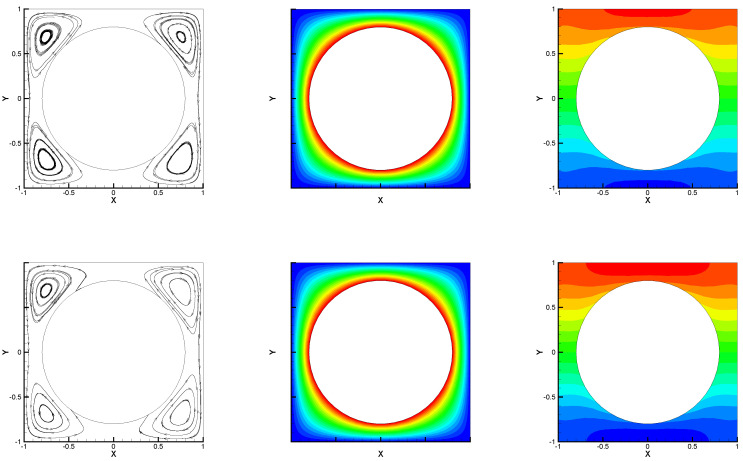
Numerical streamlines (the first column), isotherms (the second column), and isobars (the third column) for Ra=100 (the first line) and Ra=250 (the second line) with $r_{in}=0.8$.

In the second numerical test, we considered the hot cylinder problem solving the proposed algorithm with different Rayleigh numbers. The boundary conditions are given in [28,29], i.e., $\frac{\partial T}{\partial n}=1$ on inner wall, T=0 on the other wall, and zero Dirichlet condition on velocity were imposed. Set Pr=0.7,κ=1, γ=0, and $h=\frac{1}{80}$. Figure 3 and Figure 4 express the numerical streamlines, isobars, and isotherms for different radii of inner circle $r_{in}$ based on Ra=100 and Ra=250 with ρ=1.6. We observe that it shapes two vortices when $r_{in}=0.2$ and four vortices when $r_{in}=0.8$, which were found to be in good agreement with those reported in [28,29]. Therefore, the given method captured this classical model well.

In Table 3 and Table 4, we show the CPU time and the maximum value of velocity at x=0.5 and y=0.5 by Algorithms 1 and 2 with ρ=1.6 and Wang’s algorithm [29] for $r_{in}=0.2$ and $r_{in}=0.8$, respectively. From Table 3 and Table 4, we find that the proposed algorithm took the least computational time among these algorithms to obtain almost the same maximum value of velocity. In particular, Algorithm 1 did not work when Ra=250. Therefore, the proposed algorithm solved this model well.

## 5. Conclusions

In conclusion, we designed a Uzawa-type iterative algorithm based on the mixed finite element method to solve the stationary natural convection model. Compared with the common Uzawa iterative algorithm, a central feature of the proposed algorithm is that it produced weakly divergence-free velocity approximation. This algorithm can be extended to the double-diffusive natural convection [30] and the magnetohydrodynamics flows [31].

## Figures and Tables

**Table 1:** The iterative step *n* with the relaxation parameter ρ.

ρ	0.1	0.2	0.3	0.4	0.5	0.6	0.7	0.8	0.9	1.0	1.1	1.2	1.3	1.4	1.5	1.6	1.7	1.8	1.9	2.0
Algorithm 1	509	280	197	153	126	107	93	83	74	67	62	57	53	50	47	44	49	76	159	/
Algorithm 2	531	289	202	156	127	108	94	83	74	67	61	56	52	44	48	42	50	77	154	/

The mark “/” means that the iterative step was larger than 600.

**Table 2:** The value of $∥\nabla \cdot u_{h}^{n}∥$ with different Rayleigh numbers Ra.

Ra	10	100	150	180
Algorithm 2	1.82 × 10${}^{-8}$	2.65 × 10${}^{-10}$	2.02 × 10${}^{-11}$	4.96 × 10${}^{-12}$
Algorithm 1	3.50 × 10${}^{-18}$	/	/	/

The mark “/” means that the iterative step was larger than 600.

**Table 3:** Comparisons of numerical results from different algorithms with $h=\frac{1}{80},r_{in}=0.2$.

		*Ra* = 100			*Ra* = 250	
	*x* = 0.5	*y* = 0.5	CPU Time	*x* = 0.5	*y* = 0.5	CPU Time
Algorithm 2	0.281	0.284	14.135	0.755	0.760	22.135
Algorithm 1 [19]	0.263	0.465	33.772	/	/	/
Wang’s algorithm [29]	0.274	0.279	51.890	0.714	0.722	56.571

The mark “/” means that the iterative step was larger than 600.

**Table 4:** Comparisons of numerical results from different algorithms with $h=\frac{1}{80},r_{in}=0.8$.

		*Ra* = 100			*Ra* = 250	
	*x* = 0.5	*y* = 0.5	CPU Time	*x* = 0.5	*y* = 0.5	CPU Time
Algorithm 2	0.039	0.085	1.811	0.098	0.213	2.191
Algorithm 1 [19]	0.039	0.085	2.077	/	/	/
Wang’s algorithm [29]	0.039	0.086	8.851	0.098	0.214	9.169

The mark “/” means that the iterative step was larger than 600.

## Data Availability

Data sharing not applicable.
